# Four-bar Geometry is Shared among Ecologically DivergentFish Species

**DOI:** 10.1093/iob/obae019

**Published:** 2024-06-14

**Authors:** H Camarillo, E D Burress, M M Muñoz

**Affiliations:** D epartment of Ecology and Evolutionary Biology, Yale University, New Haven, CT 06510, USA; D epartment of Ecology and Evolutionary Biology, Yale University, New Haven, CT 06510, USA; Department of Biological Sciences, University of Alabama, Tuscaloosa, AL 35487, USA; D epartment of Ecology and Evolutionary Biology, Yale University, New Haven, CT 06510, USA

## Abstract

Understanding the factors that influence morphological evolution is a major goal in biology. One such factor is the ability to acquire and process prey. Prey hardness and evasiveness are important properties that can impact evolution of the jaws. Similar diets and biomechanical systems have repeatedly evolved among fish lineages, providing an opportunity to test for shared patterns of evolution across distantly related organisms. Four-bar linkages are structures often used by animals to transmit force and motion during feeding and that provide an excellent system to understand the impact of diet on morphological and biomechanical evolution. Here, we tested how diet influences the evolutionary dynamics of the oral four-bar linkage system in wrasses (Family: Labridae) and cichlids (Family: Cichlidae). We found that shifts in prey hardness/evasiveness are associated with limited modifications in four-bar geometry across these two distantly related fish lineages. Wrasse and cichlid four-bar systems largely exhibit many-to-one mapping in response to dietary shifts. Across two iconic adaptive radiations of fish, an optimal four-bar geometry has largely been co-opted for different dietary functions during their extensive ecological diversification. Given the exceptional jaw diversity of both lineages, many-to-one mapping of morphology to mechanical properties may be a core feature of fish adaptive radiation.

## Spanish

Entender los factores que influyen en la evolución morfológica es un objetivo principal en biología. Uno de esos factores es la capacidad para atrapar y procesar presas. La dureza y evasividad de las presas son propiedades importantes que pueden impactar la evolución de las mandíbulas. Dietas y sistemas biomecánicos similares han evolucionado repetidamente entre linajes de peces, proporcionando una oportunidad para evaluar patrones evolutivos compartidos entre organismos lejanamente emparentados. Los mecanismos de cuatro barras en animales, usados usualmente para transmitir fuerza y movilidad durante la alimentación, proveen un sistema excelente para entender el efecto de la dieta en la evolución morfológica y biomecánica. Aquí, evaluamos cómo la dieta influye en las dinámicas evolutivas del mecanismo oral de cuatro barras en lábridos (Familia: Labridae) y cíclidos (Familia: Cichlidae). Encontramos que los cambios en la dureza y evasividad de las presas están asociados con modificaciones limitadas de la geometría de las cuatro barras entre esos dos linajes de peces lejanamente emparentados. Los mecanismos de cuatro barras de los lábridos y cíclidos muestran en gran medida diferentes estructuras que conducen a un mismo resultado funcional en respuesta a cambios de dieta. A lo largo de dos radiaciones adaptativas icónicas de peces, una geometría óptima de cuatro barras ha sido en gran medida adaptada para diferentes funciones dietéticas durante una diversificación ecológica extensa. Dada la excepcional diversidad de mandíbulas en ambos linajes, estructuras que conducen a un mismo resultado en morfología y propiedades mecánicas podrían ser características esenciales durante la radiación adaptativa de peces.

Translated by Saúl Dominguez-Guerrero, Postdoctoral Associate- Yale University

## Portuguese

Entender os fatores que influenciam a evolução morfológica é um dos maiores objetivos na biologia. Um destes fatores é a habilidade de capturar e processar a presa. O quão dura e evasiva é a presa constituem propriedades importantes que podem impactar a evolução das mandíbulas. Dietas e sistemas biomecânicos similares evoluíram repetidamente entre as linhagens de peixes, abrindo uma oportunidade para se testar a presença de padrões evolutivos compartilhados entre organismos distantemente relacionados. Ligações de quatro barras são estruturas frequentemente usadas pelos animais para transmitir força e movimento durante a alimentação, compreendendo um excelente sistema para se entender o impacto da dieta na evolução morfológica e biomecânica. Neste trabalho testamos como a dieta influencia a dinâmica evolutiva do sistema oral de ligação de quatro barras nos peixes bodiões (família: Labridae) e ciclídeos (família: Cichlidae). Encontramos que mudanças no consumo de presas duras e evasivas estão associadas com pequenas modificações na geometria de quatro barras nessas duas linhagens de peixes distantemente relacionadas. Os sistemas de quatro barras de bodiões e ciclídeos apresentam, em grande parte, um mapeamento “muitos-para-um” em resposta a mudanças na dieta. Nestas duas icônicas radiações adaptativas de peixes, uma geometria de quatro barras ótima foi amplamente cooptada para diferentes funções na dieta

durante sua extensa diversificação ecológica. Dada a excepcional diversidade de mandíbulas de ambas as linhagens, o mapeamento “muitos-para-um” da morfologia para propriedades mecânicas pode ser uma característica central na radiação adaptativa dos peixes.

Translated by Laura Alencar, Associate Research Scientist, Yale University

## German

Das Verständnis der Faktoren, die die morphologische Evolution beeinflussen, ist ein wichtiges Ziel der Biologie. Einer solcher Faktoren ist die Fähigkeit, Beute zu erbeuten und zu verarbeiten. Zum Beispiel, die Härte und Ausweichverhalten der Beute sind wichtige Eigenschaften, die die Evolution der Kiefer eines Organismus beeinflussen können. Ähnliche Ernährungsweisen und biomechanische Systeme haben sich wiederholt in Fischlinien entwickelt, was die Möglichkeit bietet, gemeinsame Evolutionsmuster bei entfernt verwandten Organismen zu testen. Viergelenke sind Strukturen, die Tiere häufig zur Übertragung von Kraft und Bewegung bei der Nahrungsaufnahme verwenden. Viergelenke bieten ein hervorragendes System zum Verständnis der Auswirkungen der Ernährung auf die morphologische und biomechanische Entwicklung. In dieser Studie untersuchen wir, wie Ernährung die Evolutionsdynamik des oralen Viergelenksystems bei Lippfischen (Familie: Labridae) und Buntbarschen (Familie: Cichlidae) beeinflusst. Wir erfassen, dass Schwankungen in der Härte/Ausweichfähigkeit der Beute mit begrenzten Veränderungen der Viergelenkgeometrie bei diesen beiden entfernt verwandten Fischlinien verbunden sind. Viergelenksysteme von Lippfischen und Buntbarschen weisen als Reaktion auf Ernährungsumstellungen weitgehend eine Viele-zu-eins-Zuordnung auf. Wir argumentieren, dass bei zwei ikonischen adaptiven Radiationen von Fischen im Laufe ihrer umfassenden ökologischen Diversifizierung eine optimale Viergelenkgeometrie weitgehend für unterschiedliche Ernährungsfunktionen übernommen wurde. Angesichts der außergewöhnlichen Kiefervielfalt beider Linien könnte die Viele-zu-eins-Zuordnung der Morphologie zu mechanischen Eigenschaften ein zentrales Merkmal der adaptiven Radiation bei Fischen sein.

Translated by Diego-Ellis Soto, PhD Student, Yale University

## Korean

요약: 형태적 진화의 요인이 무엇인지 파악하는 것은 생물학의 주요 과제이다. 형태적 진화의 요인으로서 한 가지 예시는 먹잇감을 잡고 씹어삼키는데에 필요한 능력이다. 이는 먹잇감의 단단한 정도와 회피능력이 포식자의 턱 구조 진화에 중요한 기초가 되기 때문이다. 어류는 척추동물 중 가장 계통적 다양성이 높은 분류군으로, 비슷한 식성과 생물역학구조가 독립적으로 여러 번 진화했고, 따라서 유연관계가 상대적으로 먼 분류군 사이에서 어떠한 요인이 비슷한 진화적 결과를 만들어 내었는지를 고찰하기에 알맞은 분류군이다. 아랫턱, 코뼈, 앞위턱뼈, 및 두개골을 연결하는 해부학적 연결고리[이하 “4절링크” (“four-bar linkages”)]는 동물이 먹잇감을 씹어삼키는 과정에서 힘(force)과 운동(motion)을 전달하는 역할을 하기 때문에, 해당 구조의 형태와 생물역학구조의 진화에 식성이 어떤 영향을 미치는지 연구하기에 아주 적합한 해부학적 구조이다. 본 연구에서는 유연관계가 상대적으로 먼 놀래기과(Labridae)와 시클리드과(Cichlidae) 어류에서 4절링크의 형태적 진화에 식성이 어떤 영향을 미쳤는지를 검토하였다. 먹잇감의 단단한 정도와 회피능력이 4절링크의 형태학적 변이에 미치는 영향은 적었으며, 4절링크의 형태적 변이는 먹잇감의 종류와 다대일 매핑(many-to-one mapping)의 관계를 보였다. 적응방산으로 대표되는 두 분류군의 생태적 다양화 과정에서, 최적화 된 4절링크의 형태는 먹잇감의 다양성을 반영하였다. 구강구조의 압도적인 해부학적 다양성으로 널리 알려진 두 분류군으로부터, 저자는 형태와 생물역학적 다대일 매핑(many-to-one mapping) 관계가 어류 적응방산의 대표적인 결과의 하나임을 밝혔다.

Translated by Daemin Kim, Postdoctoral Associate, Yale University

## French

Comprendre les facteurs qui influencent l’évolution morphologique est un objectif majeur en biologie. L'un de ces facteurs est la capacité d'acquérir et de traiter des proies. La dureté et le caractère évasif des proies sont des propriétés importantes qui peuvent avoir un impact sur l'évolution des mâchoires. Des régimes alimentaires et des systèmes biomécaniques similaires ont évolué à plusieurs reprises parmi les lignées de poissons, offrant ainsi l'occasion de tester des modèles d’évolution partagés entre des organismes éloignés. Les liaisons à quatre barres sont des structures souvent utilisées par les animaux pour transmettre la force et le mouvement pendant l'alimentation, et qui constituent un excellent système pour comprendre l'impact du régime alimentaire sur l'évolution morphologique et biomécanique. Ici, nous avons testé comment le régime alimentaire influence la dynamique évolutive du système de liaison orale à quatre barres chez les labres (famille : Labridae) et les cichlidés (famille : Cichlidae). Nous avons constaté que les changements dans la dureté/le caractère évasif des proies sont associés à des modifications limitées de la géométrie à quatre barres dans ces deux lignées de poissons éloignées. Les systèmes à quatre barres de labres et de cichlidés présentent en grande partie une cartographie plusieurs-à-un en réponse aux changements

de régime alimentaire. Pour deux radiations adaptatives emblématiques de poissons, une géométrie optimale à quatre barres a été largement adoptée pour différentes fonctions alimentaires au cours de leur vaste diversification écologique. Compte tenu de la diversité exceptionnelle des mâchoires des deux lignées, la cartographie plusieurs-à-un de la morphologie aux propriétés mécaniques peut être une caractéristique essentielle du rayonnement adaptatif des poissons.

Translated by Pauline Raimondeau, Postdoctoral Associate, Yale University

## Introduction

A general goal of biology is to understand asymmetric patterns of trait evolution: Why are some traits highly diverse while others are less so? Central to this phenomenon are ecological shifts in fitness-based activities like food acquisition and processing, which can drastically reorganize the performance landscapes to which trait evolution responds (Hoffmann and Hercus 2000; Arnold 2003; Arbour et al. 2020). The study of biomechanics provides a fertile arena in which to link mechanical principles to patterns of phenotypic diversity (Vogel 2013; Taylor and Thomas 2014). Convergent evolution of similar mechanical structures provides a naturally replicated framework in which to test whether common shifts in biologically relevant motion result in shared (or different) patterns of trait evolution, and to extract generalizable principles by which biomechanical and morphological diversity evolve. Here, we examine how a common mechanical system (four-bar linkages) evolves in response to dietary shifts across distantly related fish lineages (wrasses and cichlids).

Acquiring and processing food is central to survival for organisms. One of the most important biomechanical challenges for predation relates to prey hardness. Many lineages have independently evolved the ability to dislodge and/or crush hard-bodied prey (often termed “durophagy”) (Hernandez and Motta 1997; Aguirre et al. 2003; Herrel and O'Reilly 2006; Kolmann and Huber 2009). For example, spotted hyenas have robust skulls specialized to crush bones, and their bites are among the most forceful ever recorded (Binder and Van Valkenburgh 2000; Tanner et al. 2008, 2010). By contrast, many prey are soft-bodied and, oftentimes, also evasive, imposing a different set of biomechanical demands for predators; many fish, for example, generate suction via jaw movement to rapidly draw evasive prey into their mouths (Wainwright et al. 2001). Central to dietary evolution are the biomechanical systems that assist with prey acquisition and processing. Therefore, macroevolutionary analyses of biomechanical feeding systems are well poised to reveal how diet impacts phenotypic diversification.

**Fig. 1:**
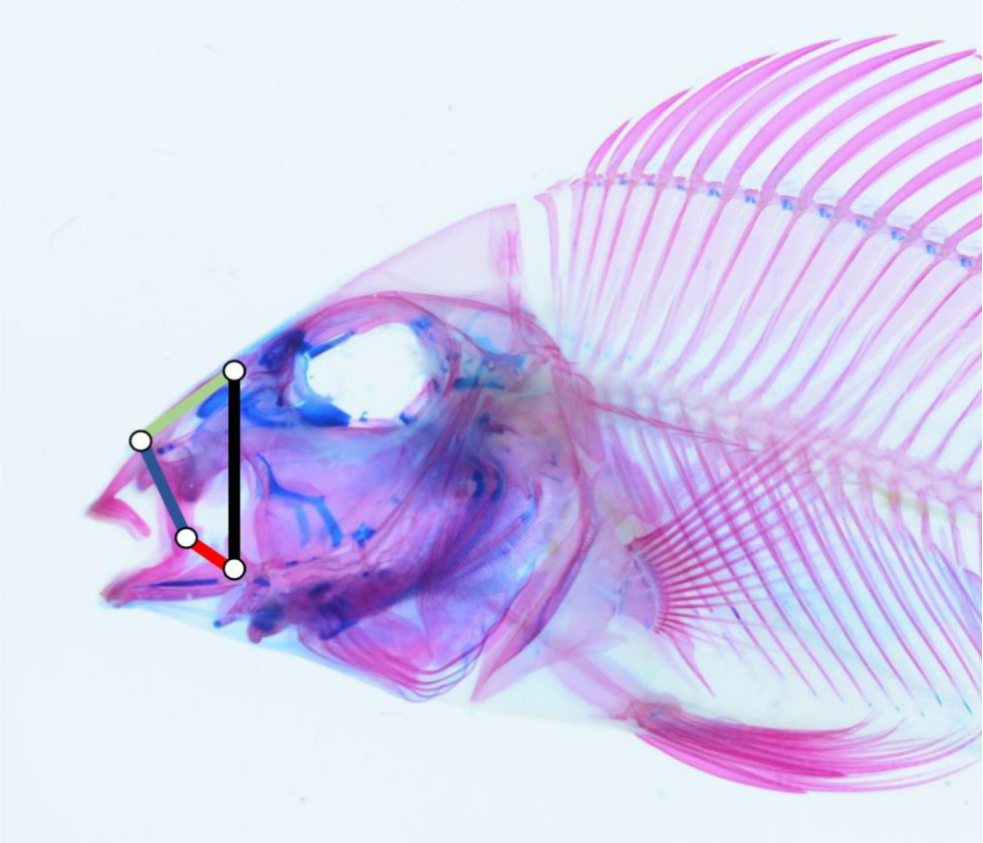
The oral four bar-linkage system in fish (wrasses and cichlids). Each color of the four-bar linkage indicates a different morphological link. Black = fixed link. Red = input link. Blue = output link. Green = coupler link. Alexus S. Roberts Hugghis assisted with taking the picture of this cleared and stained specimen of *Geophagus abalios*.

Four-bar linkages are mechanical structures that facilitate anatomical motion, including during prey acquisition and breakdown (Aerts and Verraes 1984; Westneat 1990, 1994; Muller 1996). These systems are comprised four rigid levers that interact in a chain or loop to transmit force and motion (Westneat 1994) and that have independently arisen in many animal lineages to facilitate a variety of locomotor functions (Westneat 1990; Hoese and Westneat 1996; Muller 1996; Patek et al. 2004, 2007). In many bony fish, for example, the oral four-bar linkage system is used to model premaxillary protrusion, which assists in generating suction and grasping prey (Anker 1974; Westneat 1990; Martins 1994; Muller 1996). In this four-bar system, the lower jaw serves as the input link: As it rotates, the lower jaw induces movement of the maxilla (output link) and the nasal bone (coupler), ultimately resulting in the upper jaw rotating and protruding (Fig. 1).

**Fig. 2:**
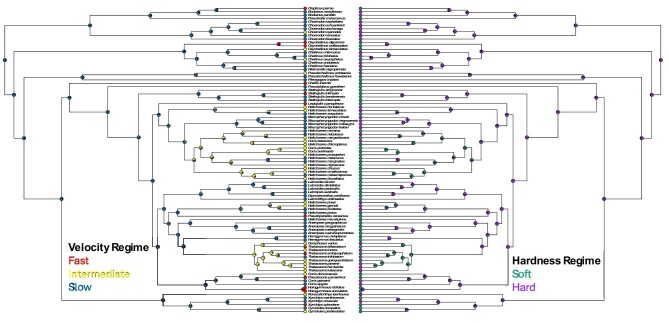
Phylogenetic tree used for wrasses (*n* = 90) with ancestral reconstructions of diet mapped. Dietary mapping based on the velocity-based regime is shown on the left and on the hardness-based regime is shown on the right.

**Fig. 3:**
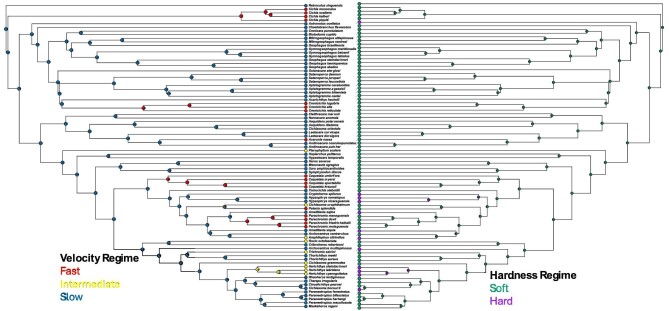
Phylogenetic tree used for cichlids (*n* = 84) with ancestral reconstructions of diet mapped. Dietary mapping based on the velocity-based regime is shown on the left and on the hardness-based regime is shown on the right.

Wrasses (Family: Labridae) and cichlids (Family: Cichlidae) represent two highly diverse fish adaptive radiations in which dietary preferences have frequently shifted, with some species specializing on relatively soft, more evasive prey like fish, and others preferring more sedentary, hard-shelled prey like mollusks, and much variation therein (Price et al. 2011; Burress 2015; Figs. 2 and 3). The similarity in prey characteristics that underlie much of the dietary diversity in these two distantly related lineages inspires comparative inquiry. Both wrasses and cichlids have a pharyngeal jaw system that does the heavy lifting of crushing hard-shelled prey (Liem and Sanderson 1986). The oral jaws, by contrast, typically participate in prey capture (Ferry-Graham et al. 2002; Wainwright et al. 2004; Wainwright and Richard 1995; 2012), and may evolve somewhat independently of the pharyngeal jaw system (e.g., Burress et al. 2020; Conith and Albertson 2021; Ronco and Salzburger 2021; Burress and Muñoz 2021; Roberts-Hugghis et al. 2023). Correspondingly, while we expect the evolution of four-bar linkage systems to generally reflect shifts in diet, the magnitude of these evolutionary shifts may vary between lineages.

Output motion of four-bar linkage systems has been most frequently described using kinematic transmission, KT, a dimensionless ratio describing angular output motion relative to angular input motion (Hulsey and Wainwright 2002; Olsen and Westneat 2016). KT is a useful mechanical property that is widely used to characterize trade-offs between force (lower KT) and velocity (higher KT) in four-bar linkages. In four-bar linkage systems, KT does not respond to link size variation equally. Instead, some levers contribute disproportionately to KT, whereas size changes in other levers can have little to no effect on four-bar motion (a phenomenon termed “mechanical sensitivity”; Anderson and Patek 2015). In the wrasse four-bar system, changes in the output link induce the greatest proportional change in KT (Anderson and Patek 2015; Muñoz et al. 2018). In wrasses, KT is also sensitive to input link variation (but not to the same degree as the output link), whereas coupler link size impacts KT only weakly (Muñoz et al. 2018). In cichlids, changes in the input link induce the greatest proportional change in KT (Muñoz et al. 2018). KT is largely insensitive to both output link and coupler link variation (i.e., mechanical sensitivity disproportionately centers around input link size) (Muñoz et al. 2018). Given its importance for emergent mechanical variation in both cichlid and wrasse four-bar systems, we predict that the strongest phenotypic shifts should occur in the output link for wrasses and the input link for cichlids, and that the magnitude of changes in the evolutionary dynamics between dietary types will correspondingly be highest for this link (as compared to the other mobile links).

Here, we integrated morphological, mechanical, dietary, and phylogenetic data for wrasses (*N* = 90 species) and cichlids (*N* = 84 species) to test how transitions in diet influence the evolutionary dynamics of the four-bar system. We first tested whether diet influenced the diversity of the four-bar linkage system among wrasses and cichlids by measuring disparity between dietary groups. We then fitted a series of evolutionary models to the morphological and dietary data to test whether (and how) diets influence the tempo and mode of trait evolution. Specifically, we tested whether transitions between different prey types (e.g., hard-bodied vs. soft-bodied) resulted in predictable changes in four-bar linkage morphology and mechanical properties, and predictable shifts in the evolutionary rate of those features. We predicted that the magnitude of phenotypic shifts would be strongest for traits exhibiting the greatest mechanical sensitivity (Muñoz et al. 2018) to most efficiently modify motion according to biomechanical demands imposed by different prey. Whether slowdowns in four-bar evolution in cichlids or wrasses is associated with less phenotypic disparity, or with shifts in the evolutionary phenotypic optimum, is not clear (but see Burress and Muñoz 2023a). In the four-bar system of the mantis shrimp raptorial appendage (Order: Stomatopoda), for example, the shift from “spearing” mantis shrimp (primarily feed on evasive prey) to “smashing” mantis shrimp (primarily feed on hard-shelled prey) resulted in a slower rate of four-bar evolution (Muñoz et al. 2017). If the same patterns apply to the oral-four bar of fish, then linkage evolution should be slower in species that consume harder shelled prey (like mollusks). Similarly, in cichlids the evolutionary rates of oral and pharyngeal jaw morphology vary among dietary ecologies (generalist, predator, grazer, and sifter), suggesting similar processes may be at play (Burress et al 2020). It is also possible that dietary transitions are not associated with evolutionary rate shifts. Such a pattern might arise, for example, because the degree of dietary specialization is not very strong or because rates of trait evolution are not tied to dietary mode. This outcome may arise because of evolutionary decoupling between prey acquisition and processing due to pharyngognathy, because many-to-one mapping dilutes selective pressures among levers, or some combination of the above (Lauder 1995; Wainwright 2007; Lautenschlager et al. 2016; Button et al. 2017). By comparing how transitions in diet influence the evolution of the four-bar system in two canonical adaptive radiations, we hope to better understand how biomechanical systems respond to extensive ecological diversification.

## Materials and methods

### Four-bar linkage data

We gathered data for the oral four-bar linkage system for 90 species of wrasses (Family: Labridae) from Wainwright et al. (2004) and Westneat et al. (2005) and for 84 species of cichlids (Family: Cichlidae) from Burress et al. (2020). We did not include parrotfish in our wrasse dataset because secondary innovations, such as the intramandibular joint, change the dynamics of the four-bar system and, therefore, challenge direct comparisons among all labrids (Price et al. 2010). The four-bar linkage data included the relative sizes of the three mobile links in each system (input, output, and coupler links), as well as the estimated KT of the system. KT is a dimensionless ratio that can be used to characterize mechanical trade-offs between transmission of force (lower KT) and velocity (higher KT) in four-bar linkages. Wrasse species that typically feed on more evasive prey like other fish, for example, have four-bar systems with higher estimated KT values when compared to the four-bar system of species that primarily fed on hard-shelled invertebrates (Westneat 1994). A caveat, however, is that KT describes only planar motion in the four-bar system, whereas feeding motion also involves non-planar motion not captured by this two-dimensional metric, particularly in wrasses, as mouth expansion involves three-dimensional motion (Olsen and Westneat 2016; Olsen et al. 2017). Nonetheless, KT has proven useful for describing general differences in the motion of four-bar systems, as planar motion is an important feature of feeding mechanics (Alfaro et al. 2004; Wainwright et al. 2005), and provides a measure that can be readily compared among distantly related organisms (Hu et al. 2017; Muñoz et al. 2018). Here, we use KT as a heuristic to describe general differences in overall motion among four-bar linkage systems, while recognizing it does not capture all aspects of biologically relevant movement. KT values for each dataset were measured statically (Wainwright et al. 2004; Westneat et al. 2005; Anderson and Patek 2015; Burress et al. 2020). Whereas dynamic measurements consider KT over the course of the entire rotation, typically focusing on minimum KT as the preferred measurement (Patek et al. 2007), static measurements use a biologically relevant set input rotation of the input link (30° in wrasses; Alfaro et al. 2005) to calculate KT.

### Dietary data

For wrasses, we gathered dietary data from previously published literature (Hobson 1974; Randall et al. 1978; Wainwright 1988; Bellwood et al. 2006; Price et al 2011) and FishBase (Froese and Pauly 2014). Most primary literature sources and FishBase included percentage amounts of different gut contents. Dietary categorizations varied between sources, but generally included the following: echinoderms, crabs and other crustaceans, polychaete worms, plant material, mollusks, fish, coral mucous, ectoparasites, foraminifera, zooplankton, detritus, and other general invertebrates (unable to classify with greater detail). For cichlids, we also summarized data from the literature (adapted from Burress 2016; Burress et al. 2019; 2020). Dietary categorizations varied between sources, but generally included piscivores, invertivores, algivores, molluscivores, herbivores, omnivores, and planktivores. Ideally, dietary data would be treated as a continuous predictor variable (e.g., using isotope data), but highly resolved diet data at large macro-evolutionary scales are lacking (Price et al. 2011; Siqueira et al. 2020). Therefore, after morphological and diet data were collected, we discretized the variable by assigning each species to different dietary regimes. Because the choice of regime is somewhat arbitrary, we ran our analyses under two different groups of modified dietary regimes. First, we discretized diet data as either “hard-bodied” or “soft-bodied” regimes. Species for which diet primarily (60% or more of diet) consisted of “hard-bodied” prey (i.e., mollusks, crabs, echinoderms, coral, and foraminiferans) fell into this category. All species for which diet could not be classified as “hard-bodied” were assigned to the “soft-bodied” diet regime. We also assigned each species to diet regimes based on general differences in the velocity of prey (slow, intermediate, or fast). Diets consisting of slow or immobile prey (e.g., mollusks, coral mucous, and algae) were assigned to the “slow” regime. Species for which diets primarily consisted of more evasive prey, such as fish and zooplankton, were categorized to the “fast” regime. Since crabs are mobile, but not as evasive/free-swimming as either fish or plankton, species that primarily feed on crabs were classified as “intermediate” for the velocity-based regime (Ferry-Graham et al. 2002). Species that had more generalist diets, or species that we could not classify as one of the two extremes based on available diet data (i.e., general invertebrates), were assigned to the “intermediate” velocity category.

### Phylogenetic data

We used the time-calibrated wrasse phylogeny of (Hodge et al. 2020) that we pruned down to the 90 species in our dataset. The wrasse phylogeny was constructed from four mitochondrial (12S, 16S, COI, and CytB) and three nuclear gene regions (RAG2, TMO4c4, and S7) with a total of 4578 base pairs (Hodge et al 2020). For cichlids, we pruned down a time-calibrated phylogeny (Burress and Tan 2017; Burress et al. 2019) to our dataset of 84 species. This tree was constructed using six mitochondrial genes (12S, 16S, COI, CytB, ND2, and ND4) and 12 nuclear gene regions (4c4, ENC1, RAG1, RAG2, S7 intron1, SH3PX3, GLYT, MYH6, PLAGL2, PTR, SREB2, and TBR1). We used the *drop.tip* function in ape (Paradis and Schliep 2019) to prune the phylogenetic trees. All the phylogenetic analyses were performed using R version 4.0.1 (R Core Development Team 2020).

### Evolutionary relationships between dietary regime and morphology

We were interested in how variation in four-bar link size and KT reflect dietary differences. To this end, we began by testing for associations between dietary regime and morphological and mechanical diversity in each four-bar system. We compared how KT and each mobile link (input, output, and coupler) varied between dietary regimes using phylogenetic analysis of variance (ANOVA) with a residual randomization permutation procedure (Collyer and Adams 2018) in the R package geomorph. To determine the statistical significance of the comparison, we ran the model for 10,000 permutations. We confirmed statistical significance using pairwise comparisons with the *pairwise* function in the residual randomization in permutation procedures (RRPP) package (Collyer and Adams 2018).

To visually compare differences in trait space, we created 3D phylomorphospace plots using the *phylomorphospace3d* function in phytools (Revell 2012). We used size-corrected values for each mobile link to generate the phylomorphospace plot and then distinguished variation in KT and dietary regime with different colors. We then tested for significant differences in morphological disparity between regimes using the *morphol.disparity* function in the R package geomorph (Adams et al. 2024).

### Comparing the evolutionary dynamics of four-bar linkage systems among dietary regimes

We were also interested in determining whether four-bar linkage evolution (in rate and evolutionary optima) reflects diet among cichlids and wrasses. To compare the evolutionary dynamics between dietary regimes, we fitted a series of evolutionary models to the morphological, mechanical, and dietary data. To this end, we began by reconstructing the evolutionary history of dietary regimes using the *make.simmap* function in the R package phytools (Revell 2012). We did so by constructing 500 stochastic character maps to sample evolutionary changes in dietary regimes using either a transition model of equal rates (ER) or an all-rates different model (ARD) depending on which was the better fitting model for each regime (Huelsenbeck et al. 2003). An ER model best fit the hardness based regime in wrasses and the velocity based regime in cichlids. An ARD model best fit the velocity-based regime in wrasses and the hardness-based regime in cichlids. We then fitted five different models of evolution to each trait (the three mobile links and KT) across the different discrete diet histories using the R package OUwie (Beaulieu et al. 2012). We determined best fit between different Brownian motion (BM) and Ornstein–Uhlenbeck (OU) models of trait evolution using size-corrected AIC score (AIC_C_) and AIC_C_ weights (Burnham et al. 2002; 2011).

BM models assume trait evolution proceeds via a random walk such that phenotypic differences among species are proportionate to time since divergence (Felsenstein 1985). By contrast, OU models constrain evolution via the presence of one or more adaptive peaks (Hansen 1997; Butler and King 2004). Differences between models rely on variation between different parameters important for trait evolution: σ^2^ is the rate of stochastic character evolution, θ is the evolutionary optimal trait value, and α is the strength of selection towards the optimum. The simplest model we applied in OUwie, BM1, is a single-rate (σ^2^) BM model in which all species have the same rate of trait evolution. BMS is a two-rate BM model that permits the evolutionary rate to differ between dietary regimes. OU1 is an OU model characterized by a single adaptive peak (θ) for the entire group. OUM is an OU model in which separate phenotypic optima (θ) are fitted to each dietary regime. OUMV is an OU model that allows both θ and σ^2^ to vary between dietary regimes. We also included two OUM models and two OUMV models (one for the hardness-based dietary categorization and for the velocity-based dietary categorization). After fitting each model separately for each trait, we used sample size corrected AIC_C_ scores and AIC_C_ weights to compare the best fitting evolutionary models for each trait. Any models with ΔAIC_C_ ≤ 2 were considered to have equivalent support (Burnham and Anderson 2004).

Complex OU models can often be incorrectly favored over simpler models if the statistical power of the analysis is weak, for example, when the number of species sampled is relatively low (Ho and Ané 2014; Cooper et al. 2016). The OUMVA model in the OUwie package is the most complex, as it allows the phenotypic optima (θ), rate of evolution (σ^2^), and strength of selection (α) to vary among dietary regimes. Incorrect support can be particularly pronounced when fitting the OUMVA model to trait data, as accurate estimation of σ^2^ under different estimates of the α parameter is difficult (Ho and Ané 2014; Cooper et al. 2016). To assess whether we had enough statistical power to accurately fit and compare the six models available in OUwie (BM1, BMS, OU1, OUM, OUMV, and OUMVA), we simulated data for each model using the function *OUwie.sim* in the R package OUwie. Simulated data were then run through all six models in OUwie to determine if the data and parameters could be recovered. We determined that we lacked statistical power to adequately fit the OUMVA model to our trait data for wrasses and cichlids (Table S1), so chose to exclude this model from our analyses.

## Results

### Relationships between dietary mode and four-bar linkage morphology/mechanical properties in wrasses and cichlids

When comparing differences between dietary regimes, oral four-bar KT was higher in wrasses that consume more evasive prey (*P* < 0.001; Table 1A), corresponding to relatively greater feeding velocity. This change in KT correspondingly reflects shifts in four-bar geometry: In wrasses that consume more evasive prey, we also observed increases in input link length (*P* = 0.02; Table 1B) and coupler link length (*P* = 0.01; Table 1D) relative to wrasses that consume slow prey. Output link size differed among dietary regimes in wrasses (*P* = 0.03; Table 1C), but based on RRPP, we did not detect any significant pairwise differences (*P* = 0.53; Table 1C). When discretized into hardness-based regimes, we also detected reductions in KT (*P* < 0.01; Table 1A) with species that consumed hard prey having a lower KT than those that consume soft prey; however based on RRPP, we did not detect any significant pairwise differences (*P* = 0.17; Table 1A). We detected reductions in input link length for wrasses that consumed hard-bodied compared to soft bodied prey (*P* = <0.01; Table 1B). There were no differences among hardness-based regimes for the output link or the coupler link. When comparing differences in morphological disparity, we did not detect any significant differences in the velocity-modified or hardness-modified regimes in wrasses (Table 2).

**Table 1:** Summary of the results from the Phylogenetic ANOVA and the RRPP for wrasses under three dietary regimes (fast/intermediate/slow) and two dietary regimes (hard/soft)

Trait	Regime	Analysis	*F*-value	Z-score	*P*-value
(A) Kinematic transmission	Velocity	Phylogenetic ANOVA	9.87	3.33	**<0.001**
		RRPP (fast:slow)		1.80	**0.03**
		RRPP (intermediate:slow)		1.65	**0.04**
		RRPP (fast:intermediate)		–0.08	**0.55**
	Hardness	Phylogenetice ANOVA	16.9	3.05	**<0.001**
		RRPP		1.00	0.17
(B) Input link	Velocity	Phylogenetic ANOVA	5.12	2.33	**0.01**
		RRPP(fast:slow)		1.86	**0.02**
		RRPP (intermediate:slow)		0.16	0.46
		RRPP (fast:intermediate)		1.18	0.12
	Hardness	Phylogenetic ANOVA	19.77	3.30	**<0.001**
		RRPP		1.81	**0.03**
(C) Output link	Velocity	Phylogenetic ANOVA	3.79	1.84	**0.03**
		RRPP (fast:slow)		–0.05	0.53
		RRPP (intermediate:slow)		–0.05	0.53
		RRPP (fast:intermediate)		–1.33	0.89
	Hardness	Phylogenetic ANOVA	2.91	1.29	0.10
(D) Coupler link	Velocity	Phylogenetic ANOVA	5.45	2.39	**0.01**
		RRPP (fast:slow)		2.25	**0.01**
		RRPP (intermediate:slow)		−0.84	0.78
		RRPP (fast:intermediate)		2.20	**0.01**
	Hardness	Phylogenetic ANOVA	3.52	1.50	0.07

Pairwise comparison for RRPP is only reported when significant results were detected based on the phylogenetic ANOVA. Significant comparisons are denoted with bolded *P*-values.

**Table 2:** Summary of the results for the morphological disparity analyses for wrasses. There is no significant difference in morphological disparity among dietary categories for either the velocity- or hardness-based regimes

Trait	Regime	Disparity		*P*-values
Input link	Velocity	Fast Intermediate Slow	0.078 0.070 0.071	Fast Intermediate Slow 1.000 0.2681.000. 0.3370.7491.00
	Hardness	Hard Soft	0.057 0.063	Hard 1.00 0.327
Output link	Velocity	Fast Intermediate Slow	0.061 0.059 0.065	Fast Intermediate Slow 1.000 0.7711.000 0.6290.2481.000
	Hardness	Hard Soft	0.062 0.064	Hard 1.00 0.76
Coupler link	Velocity	Fast Intermediate Slow	0.089 0.081 0.083	Fast Intermediate Slow 1.000 0.4681.000 0.5350.7731.000
	Hardness	Hard Soft	0.101 0.099	Hard 1.00 0.715

Unlike the wrasses, we did not detect any significant differences in oral four-bar KT of cichlids when comparing the velocity-based dietary regime (*P* = 0.31; Table 3A) or the hardness-based regime (*P* = 0.06; Table 3A). We detected differences in the input link when comparing between the hardness regime (*P* = 0.03; Table 3B), but after the RRPP analysis there was no significant pairwise difference (*P* = 0.37; Table 3B). We detected no differences in the output link for the velocity-based regime (*P* = 0.24; Table 3C) or the hard based regime (*P* = 0.32; Table 3C). The only significant difference was in the coupler link with the velocity-based regime (*P* < 0.01; Table 3D). The RRPP analysis also indicated pairwise differences between cichlids that consume fast prey and cichlids that consume slow prey (*P* = 0.03; Table 3D). In this case, cichlids that eat slower prey have a larger coupler link when compared with those that eat faster prey. When comparing differences in morphological disparity, we did not detect any significant differences in the velocity-modified or hardness-modified regimes in cichlids (Table 4).

**Table 3:** Summary of the results from the phylogenetic ANOVA and the RRPP for cichlids under three dietary regimes (fast/intermediate/slow) and two dietary regimes (hard/soft)

Trait	Regime	Analysis	*F*-Value	Z-Score	*P*-value
(A) Kinematic transmission	Velocity	Phylogenetic ANOVA	1.18	0.5304	0.31
	Hardness	Phylogenetice ANOVA	3.75	1.53	0.06
(B) Input link	Velocity	Phylogenetic ANOVA	0.68	0.03	0.50
	Hardness	Phylogenetic ANOVA	5.97	1.89	**0.03**
		RRPP		0.38	0.37
(C) Output link	Velocity	Phylogenetic ANOVA	1.45	0.73	0.24
	Hardness	Phylogenetic ANOVA	0.96	0.52	0.32
(D) Coupler link	Velocity	Phylogenetic ANOVA	5.71	2.52	**0.004**
		RRPP (fast:slow)		1.82	**0.03**
	Hardness	Phylogenetic ANOVA	1.62	0.87	0.20

Pairwise comparison for RRPP is only reported when significant results were detected based on the phylogenetic ANOVA. Significant comparisons are denoted with bolded *P*-values.

**Table 4:** Summary of the results for the morphological disparity analyses for cichlids. There is no significant difference in morphological disparity among dietary categories for either the velocity- or hardness-based regimes

Trait	Regime	Disparity		*P*-values
Input link	Velocity	Fast intermediate slow	0.068 0.078 0.071	Fast Intermediate Slow 1.000 0.5831.000. 0.8420.6141.00
	Hardness	Hard soft	0.082 0.076	Hard 1.00 0.381
Output link	Velocity	Fast Intermediate Slow	0.064 0.053 0.045	Fast Intermediate Slow 1.000 0.4031.000 0.0700.3281.000
	Hardness	Hard Soft	0.044 0.048	Hard 1.00 0.32
Coupler link	Velocity	Fast Intermediate Slow	0.165 0.115 0.126	Fast Intermediate Slow 1.000 0.0761.000 0.0820.6041.000
	Hardness	Hard Soft	0.132 0.49	Hard 1.00 0.193

### Dietary regime impacts four-bar linkage evolution in wrasses and cichlids

We found strong support for transitions in wrasse diet being associated with different four-bar morphology and shifts in the rate of four-bar evolution (Table 5A). The best-supported model for the evolution of wrasse oral four-bar KT and the output link was a multi-peak, multi-rate model with the velocity-based regimes (OUMV; Table 5C). For the input link, a single-rate, single peak OU model is sufficient to explain differences between dietary categories (**OU1**; Table 5B). For the coupler link, a two-rate BM model is sufficient to explain phenotypic differences among wrasses, and is robust to all dietary classifications (BM1; Table 5D). For KT, wrasses that primarily fed on faster prey had a higher optimal trait value than those that consumed intermediate and slower prey (Table 6). For the output link, wrasses that fed on that faster prey had a lower optimal trait value than those that consumed intermediate or slower prey (Table 6). The evolutionary rate for KT was twice as fast for wrasses that feed on fast prey compared with those that feed on slow prey and intermediate prey. For the input link, the evolutionary rate was comparable for wrasses that feed on fast and slow prey. For the output link, the evolutionary rate for wrasses that consumed fast prey was half as fast as those that consumed slow prey and in the intermediate velocity category.

**Table 5:** Summary of model fits for KT and each mobile component of the four-bar system (input, output, and coupler links) in wrasses under different dietary regimes

Trait	Model	ΔAIC_C_	Weight
(A) Kinematic transmission	BM1	16.45	0.00
	BMS	13.31	0.00
	OU1	3.06	0.12
	OUM_Velocity_	2.60	0.16
	OUM_Hardness_	3.66	0.09
	**OUMV_Velocity_**	**0.00**	**0.57**
	OUMV_Hardness_	4.78	0.05
(B) Input link	BM1	14.31	0.00
	BMS	16.63	0.00
	**OU1**	**1.51**	**0.16**
	**OUM_Velocity_**	**0.00**	**0.35**
	**OUM_Hardness_**	**1.76**	**0.14**
	**OUMV_Velocity_**	**1.04**	**0.21**
	**OUMV_Hardness_**	**1.82**	**0.14**
(C) Output link	BM1	16.33	0.00
	BMS	4.29	0.09
	OU1	5.28	0.06
	OUM_Velocity_	7.08	0.03
	OUM_Hardness_	6.68	0.03
	**OUMV_Velocity_**	**0.00**	**0.79**
	OUMV_Hardness_	8.03	0.01
(D) Coupler link	**BM1**	**1.17**	**0.15**
	**BMS**	**1.12**	**0.16**
	OU1	3.31	0.05
	**OUM_Velocity_**	**1.25**	**0.15**
	**OUM_Hardness_**	**1.36**	**0.14**
	OUMV_Velocity_	2.57	0.08
	**OUMV_Hardness_**	**0.00**	**0.28**

The best fitting model is given with ΔAIC_C_ of 0 and models with equivalent support (ΔAIC_C_ ≤ 2) are shown in bold. AIC weight is also given. Note that we ran analyses with different dietary categories corresponding to different dietary regimes for OUM and OUMV models (see Methods).

**Table 6:** Summary of mean optimal trait value (θ) for the coupler link in cichlids and the input link, output link, and KT in wrasses, since there was strong support for a multi-peak OU model. The evolutionary rate is also shared for wrasses because a two-rate model was strongly supported

Group and Diet Regime	Trait	Mean θ	Mean σ^2^
Cichlid (3 regime; fast:intermediate:slow)	Coupler	(−0.375, −0.278, −0.269)	
Wrasse (3 regime; fast:intermediate:slow)	KT	(−0.01, −0.061, −0.133)	(0.002, 0.001, 0.001)
	Output	(−0.327, −0.315, −0.282)	(0.001, 0.0001, 0.0002)

In cichlids, the best-supported model for the oral four-bar KT, the input link, and the output link was a single-rate, single-peak OU model (OU1; Table 7). The best-supported model for the coupler link was a two-peak, single-rate model for the velocity-based regime (OUM; Table 7D). The evolutionary optimum for the coupler link was smaller in cichlids that consumed faster prey than slower prey (Table 6).

**Table 7:** Summary of model fits for KT and each mobile component of the four-bar system (input, output, and coupler links) in cichlids under different dietary regimes

Trait	Model	ΔAIC_C_	Weight
(A) Kinematic Transmission	BM1	44.27	0.00
	BMS	44.56	0.00
	**OU1**	**0.00**	**0.36**
	OUM_Velocity_	2.28	0.12
	OUM_Hardness_	2.05	0.13
	**OUMV_Velocity_**	**0.02**	**0.36**
	OUMV_Hardness_	4.17	0.05
(B) Input link/lower jaw	BM1	44.76	0.00
	BMS	46.63	0.00
	**OU1**	**0.00**	**0.45**
	**OUM_Velocity_**	**0.57**	**0.34**
	**OUM_Hardness_**	**1.93**	**0.17**
	OUMV_Velocity_	7.14	0.01
	OUMV_Hardness_	5.19	0.03
(C) Output link/maxilla	BM1	17.07	0.00
	BMS	13.04	0.00
	**OU1**	**0.00**	**0.48**
	OUM_Velocity_	2.66	0.13
	**OUM_Hardness_**	**1.85**	**0.19**
	OUMV_Velocity_	2.73	0.12
	OUMV_Hardness_	3.50	0.08
(D) Coupler link/nasal	BM1	20.88	0.00
	BMS	20.42	0.00
	OU1	6.32	0.03
	**OUM_Velocity_**	**0.00**	**0.71**
	OUM_Hardness_	7.44	0.02
	OUMV_Velocity_	2.27	0.23
	OUMV_Hardness_	7.94	0.01

The best fitting model is given with ΔAIC_C_ of 0 and models with equivalent support (ΔAIC_C_ ≤ 2) are shown in bold. AIC weight is also given. Note that we ran analyses with different dietary categories corresponding to different dietary regimes for OUM and OUMV models (see Methods).

## Discussion

Wrasses and cichlids are iconic fish adaptive radiations, known for their extensive diversity of jaw morphologies and feeding ecologies (Price et al. 2011). We found that dietary transitions in wrasses and cichlids resulted in different outcomes for the biomechanical evolution of the jaws, perhaps as a consequence of their more than 100 million year divergence (Ghezelayagh et al. 2022). In both cases, four-bar geometry evolved in lineages to accommodate different demands for either greater force (lower KT) in species that consume slower, harder prey, or to faster velocity (higher KT) in species that consume softer, faster prey. For wrasses, our results were consistent with those of previous work (Anderson and Patek 2015; Muñoz et al. 2018), and we found that shifts in four-bar morphology were biased toward the most mechanically sensitive trait (output link). However, corresponding shifts in morphology were not biased to mechanically sensitive traits in cichlids. Instead, shifts in four-bar morphology to accommodate changes in diet involve subtle changes in multiple linkages. Moreover, we found that different species with different dietary categories overlap in four-bar morphospace, indicating that similar oral four-bar configurations are viable across a range of dietary ecologies. We unpack these findings in greater detail below.

**Fig. 4:**
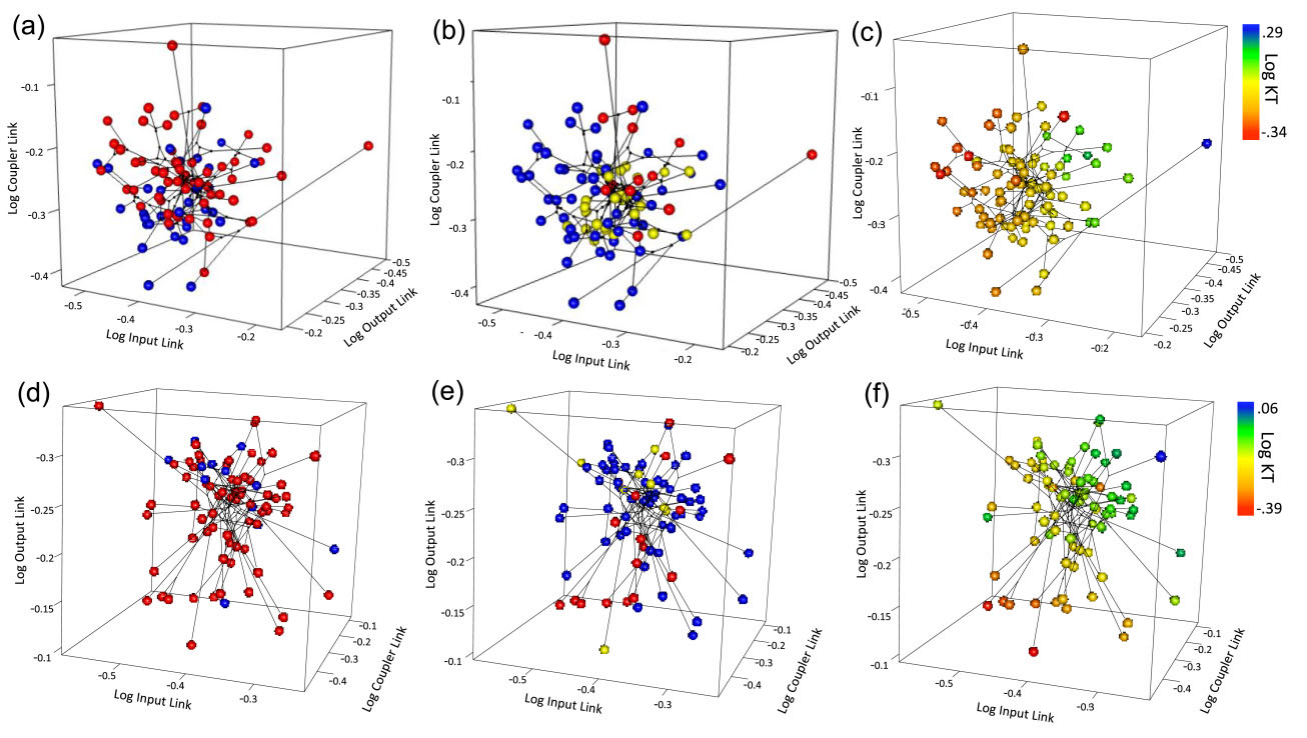
Each plot is a 3D phylomorphospace showing variation (as ratio relative to fixed link) in input link, output link, and coupler link length for wrasses (A + B + C) and cichlids (D + E + F). The first column illustrates morphospace for species that feed on hard (blue) and soft (red) prey. The second column illustrates morphospace for species that feed on slow (blue), intermediate (yellow), and fast (red) prey. The third column indicates differences in KT. Both lineages largely overlap in trait space between dietary regimes (both for the hardness-based regimes and velocity-based regimes).

In contrast to our predictions, the model selection approach did not indicate any differences in the evolutionary rate associated with shifts between soft-bodied, evasive prey, and hard-bodied slow prey in cichlids as it did in wrasses. The best supported model for KT and the output link in wrasses was a multi-peak multi-rate model for the velocity-based regime. In cichlids a single-peak, single-rate model was sufficient to describe four-bar evolution. In both groups, but especially cichlids, species appear to largely co-opt four-bar configurations for many different feeding ecologies (Fig. 4). By contrast, previous research that has focused on jaw functional morphology has been strongly correlated with changes in rates of trait evolution (Burress and Muñoz 2023a; Arbour et al. 2020; Burress et al. 2023). This discrepancy is likely driven by many-to-one mapping (Wainwright et al. 2005; Wainwright 2007) in which different morphologies produce similar mechanical properties, a phenomenon known to be widespread in labrids and cichlids (Burress and Muñoz 2023a; Alfaro et al. 2005; Burress et al. 2023). The one exception is that shifts in diet are associated with shifts in evolutionary optima for the coupler link in cichlids.

One potential explanation for relatively weak associations between diet and four-bar evolution in wrasses centers around the diverse ecologies associated with prey capture/processing in these organisms. For example, *Choerodon schoenleinii* and *Cheilinus fasciatus* both utilize tools to crack prey open and gain access to their soft tissues (Fricke 1971; Jones et al. 2011). In these cases, tool use behavior could influence phenotypic evolution by reducing the pressure to modify the oral jaws (Wake et al. 1983; Brandon 1988; Duckworth 2009; Muñoz 2022). By contrast, species that do not utilize tools might experience stronger selection on oral jaw morphology according to mechanical properties of prey.

One potential explanation for the weak relationship between diet and four-bar evolution in cichlids when compared to wrasses could be due to differences in habitat use. Unlike wrasses, which are found mostly in coral reef habitats (Price et al. 2011), cichlids can be found in a range of habitats that vary in their complexity, such as lakes, rivers, and floodplains. Colonization of different habitat types helped facilitate speciation in cichlids due to variation across several environmental/ecological axes, including predation pressure, exploitation of the benthic-pelagic axis, and variation in prey availability (Seehausen and Wagner 2014; Burress and Tan 2017; Burress et al. 2023; Burress and Muñoz 2023b). Even if similar feeding behaviors have evolved convergently between these habitats in cichlids, differences in prey (even within the same discrete classification) can vary (Burress et al. 2013; Burress 2015; Burress et al 2019). For example, even though mollusks are hard-bodied prey, the functional demand imposed by different prey (e.g., bivalves vs. snails) could require different morphological configurations and mechanical properties (Burress et al 2013; Burress 2015). In other words, there may be finer scale variation that our broad diet categories do not characterize (see below).

The demands associated with feeding on hard- vs. soft-bodied prey present similar ecological constraints between wrasses and cichlids. In both these systems, constraints on prey processing could be partially liberated by the functional and evolutionary independence among the oral and pharyngeal jaws (Liem 1973; Liem and Sanderson 1986; Hulsey et al. 2006; Wainwright 2007; Burress and Muñoz 2021). Theory predicts that, by functionally decoupling prey capture and processing, the pharyngeal jaw apparatus can circumvent constraints imposed by biomechanical trade-offs (Wainwright 2007; Muñoz 2019). To this end, wrasses utilizing different prey items do not occupy unique regions of morphospace; rather, four-bar geometries largely overlap among different dietary modes. It is important to note that the species used in this study possess a highly modified pharyngeal jaw system (pharyngognathy), but lack secondary innovations (e.g., intramandibular joint, coalesced premaxillary teeth, and the pharyngeal jaw mill) that are present in parrotfishes (Wainwright and Price 2018). In both systems that we studied, modified pharyngeal jaws assist in crushing prey (Liem and Sanderson 1986; Wainwright et al. 2012); therefore, the force-based performance demands on the oral system likely center more on plucking, biting, or un-encrusting relatively hard-bodied, sedentary prey.

The weak relationship between diet and four-bar geometry could also be a consequence of the way we discretized our dietary categories. Previous work has used broad dietary categorizations to understand the relationship between diet and the evolution of feeding morphology and has been quite informative in vertebrates (Price and Hopkins 2015; Borstein et al. 2017; Felice et al. 2019; Arbour et al. 2020). Nevertheless, it is possible that our categories are too coarse to capture KT variation in response to diet, as feeding ecology is known to underly the diversity of cichlid jaw mechanics (Martinez et al. 2018; Burress et al. 2020). For example, in our dietary categorizations, *Crenicichla* are described as feeding on soft, evasive prey, but this clade of cichlids are pursuit predators that use ram-feeding rather than suction feeding with rapid jaw movements like ambush predators (e.g., *Petenia; Caquetaia*; Wainwright et al. 2001). In other words, there is considerable fine-scale diversity within our feeding categories that could obfuscate the dietary signal in four-bar geometry.

Furthermore, the oral four-bar linkage system provides a relatively limited snapshot into the biomechanics of prey capture in fish. This four-bar system is part of a bigger, more multidimensional feeding apparatus. For example, in other fish systems, jaw feeding systems can be more accurately described by a 17-bar system, which better captures the three-dimensional aspects of feeding (beyond the planar motion described here) (Olsen et al. 2020). Whether the relationship between diet and four-bar geometry translates when looking at other levers in the feeding apparatus in more detail remains to be investigated.

### Conclusions

Central to the fields of comparative physiology and biomechanics is discovering general rules guiding how organisms interact with their environments, and how those interactions scale up to evolutionary patterns of diversity. Many distantly related organisms independently acquired the ability to capture and process similar prey; yet, whether parallel ecological shifts should be matched by parallel outcomes in biomechanical and morphological evolution is unclear (reviewed in Muñoz 2019). In general, there was a greater effect of dietary transitions on four-bar evolution in wrasses compared with cichlids, with a single-rate, single-peak model best-fitting most traits in cichlids and a multi-peak, single-rate model best fitting most traits in wrasses, likely reflecting the importance of many-to-one mapping. There was a large amount of phenotypic overlap among species (Fig. 4), regardless of prey type, reflecting macroevolutionary “co-opting” of four-bar geometry among species with different diets. The presence of the pharyngeal jaw system in ray-finned fish may have liberated the oral jaws from some of the mechanical pressures associated with dietary specialization, highlighting the importance of historical factors among lineages in nuancing phenotypic evolution. To more accurately describe the role that transitions in diet can play in the evolutionary dynamics of trait evolution, further studies must be done both in other four-bar systems and other types of feeding systems (Meloro et al. 2011; Figueirido et al. 2013; Collar et al. 2014; Meloro et al. 2015; Hu et al. 2017; Muñoz et al. 2018). As more detailed ecological, biomechanical, and morphological datasets become available (Chang and Alfaro 2016; Muñoz and Price 2019; Siqueira et al. 2020), this goal is increasingly within reach.

## Supplementary Material

obae019_Supplemental_File
