# Supplementary material for: Four-bar Geometry is Shared among Ecologically DivergentFish Species
Source: Integr Org Biol. 2024 Jun 14;6(1):obae019. doi: 10.1093/iob/obae019 (PMC11211069; doi:10.1093/iob/obae019)
Supplement: obae019_Supplemental_File [file obae019_supplemental_file.docx]

Supplementary Table 1: Summary of model selection for wrasses and cichlids using simulated data generated using *OUwie.sim* in the R package OUwie (Beaulieu et al. 2012). Model source refers to the evolutionary model used to simulate data. The best fitting model should be the same as the model source. However, OUMVA is incorrectly favored, which indicates that OUMVA should not be included in our analyses.

| Taxa | Model Source | Model Fitted | ΔAIC_C_ | Weight |
| --- | --- | --- | --- | --- |
| Wrasses | BM1 | BM1 | 39.92 | 2.15x10^-9^ |
|  |  | BMS | 41.10 | 1.19 x10^-9^ |
|  |  | OU1 | 39.42 | 2.76 x10^-9^ |
|  |  | OUM | 37.63 | 6.73 x10^-9^ |
|  |  | OUMV | 37.86 | 6.03 x10^-9^ |
|  |  | OUMVA | 1.00 | 1.00 |
|  |  |  |  |  |
|  | BMS | BM1 | 15.08 | 5.32x10^-4^ |
|  |  | BMS | 15.58 | 4.14 x10^-4^ |
|  |  | OU1 | 17.15 | 1.89 x10^-4^ |
|  |  | OUM | 18.94 | 7.70 x10^-5^ |
|  |  | OUMV | 19.47 | 5.92 x10^-4^ |
|  |  | OUMVA | 0.000 | 9.99 x10^-1^ |
|  |  |  |  |  |
|  | OU1 | BM1 | 1.88x10^9^ | 0 |
|  |  | BMS | 1.88x10^9^ | 0 |
|  |  | OU1 | 1.88x10^9^ | 0 |
|  |  | OUM | 1.88x10^9^ | 0 |
|  |  | OUMV | 1.88x10^9^ | 0 |
|  |  | OUMVA | 0 | 1 |
|  |  |  |  |  |
|  | OUM | BM1 | 1.71x10^11^ | 0 |
|  |  | BMS | 1.71x10^11^ | 0 |
|  |  | OU1 | 1.71x10^11^ | 0 |
|  |  | OUM | 1.71x10^11^ | 0 |
|  |  | OUMV | 1.71x10^11^ | 0 |
|  |  | OUMVA | 0 | 1 |
|  |  |  |  |  |
|  | OUMV | BM1 | 2.17x10^11^ | 0 |
|  |  | BMS | 2.17x10^11^ | 0 |
|  |  | OU1 | 2.17x10^11^ | 0 |
|  |  | OUM | 2.17x10^11^ | 0 |
|  |  | OUMV | 2.17x10^11^ | 0 |
|  |  | OUMVA | 0 | 1 |
|  |  |  |  |  |
|  | OUMVA | BM1 | 5.01x10^10^ | 0 |
|  |  | BMS | 5.01x10^10^ | 0 |
|  |  | OU1 | 5.01x10^10^ | 0 |
|  |  | OUM | 5.01x10^10^ | 0 |
|  |  | OUMV | 5.01x10^10^ | 0.26 |
|  |  | OUMVA | 0 | 0.74 |
|  |  |  |  |  |
| Cichlids | BM1 | BM1 | 25.80 | 2.49x10^-6^ |
|  |  | BMS | 24.23 | 5.48 x10^-6^ |
|  |  | OU1 | 27.96 | 8.50 x10^-7^ |
|  |  | OUM | 29.92 | 4.50 x10^-7^ |
|  |  | OUMV | 26.92 | 1.43 x10^-6^ |
|  |  | OUMVA | 0 | 1 |
|  |  |  |  |  |
|  | BMS | BM1 | 31.22 | 1.66 x10^-7^ |
|  |  | BMS | 31.96 | 1.15 x10^-7^ |
|  |  | OU1 | 33.37 | 5.66 x10^-8^ |
|  |  | OUM | 35.44 | 2.01 x10^-8^ |
|  |  | OUMV | 36.19 | 1.38 x10^-8^ |
|  |  | OUMVA | 0 | 1 |
|  |  |  |  |  |
|  | OU1 | BM1 | 52.36 | 4.28 x10^-12^ |
|  |  | BMS | 52.89 | 3.27 x10^-12^ |
|  |  | OU1 | 27.75 | 9.43x10^-7^ |
|  |  | OUM | 29.89 | 3.23 x10^-7^ |
|  |  | OUMV | 30.87 | 1.98 x10^-7^ |
|  |  | OUMVA | 0 | 1 |
|  |  |  |  |  |
|  | OUM | BM1 | 91.86 | 1.12x10^-20`^ |
|  |  | BMS | 82.30 | 1.34 x10^-18^ |
|  |  | OU1 | 21.21 | 1.34 x10^-5^ |
|  |  | OUM | 9.83 | 7.28 x10^-3^ |
|  |  | OUMV | 13.08 | 1.43 x10^-3^ |
|  |  | OUMVA | 0 | 1 |
|  |  |  |  |  |
|  | OUMV | BM1 | 97.76 | 3.59 x10^-22^ |
|  |  | BMS | 99.01 | 1.93 x10^-22^ |
|  |  | OU1 | 21.76 | 1.15 x10^-5^ |
|  |  | OUM | 1.10 | 0.35 |
|  |  | OUMV | 0 | 0.61 |
|  |  | OUMVA | 5.48 | 0.04 |
|  |  |  |  |  |
|  | OUMVA | BM1 | 114.48 | 1.38 x10^-25^ |
|  |  | BMS | 112.31 | 4.10 x10^-25^ |
|  |  | OU1 | 73.11 | 1.33 x10^-16^ |
|  |  | OUM | 72.94 | 1.45 x10^-16^ |
|  |  | OUMV | 73.77 | 9.58 x10^-17^ |
|  |  | OUMVA | 0 | 1 |

Supplementary Table 2: Summary of four-bar linkage information and dietary categorizations for wrasses. Four-bar data were taken from Wainwright et al. (2004) and Westneat et al. (2005). Dietary data were collected from Randall (1967), Wainwright (1988), Bellwood et al. (2006), Price et al. (2011), Hobson (1974), Randall et al. (1978), and Fishbase (Froese and Pauly 2019). Details on how dietary categorizations were assigned can be found in the Methods.

| Species | KT | Input | Coupler | Output | Dietary Categorization | Velocity-Based Regime | Hard-Based Regime |
| --- | --- | --- | --- | --- | --- | --- | --- |
| *Anampses caeruleopunctatus* | 0.47 | 0.37 | 0.64 | 0.64 | Zoobenthos | Slow | Soft |
| *Anampses geographicus* | 0.55 | 0.29 | 0.67 | 0.52 | Zoobenthos | Slow | Soft |
| *Anampses meleagrides* | 0.54 | 0.34 | 0.64 | 0.6 | Zoobenthos | Slow | Soft |
| *Anampses neoguinaicus* | 0.47 | 0.31 | 0.57 | 0.58 | Zoobenthos | Slow | Soft |
| *Bodianus loxozonus* | 0.74 | 0.41 | 0.42 | 0.56 | Mollusk | Slow | Hard |
| *Bodianus mesothorax* | 0.75 | 0.44 | 0.38 | 0.56 | Mollusk | Slow | Hard |
| *Bodianus perditio* | 0.62 | 0.4 | 0.39 | 0.62 | Mollusk | Slow | Hard |
| *Cheilinus chlorourus* | 0.92 | 0.43 | 0.48 | 0.48 | Echinoderm & Crab | Slow | Hard |
| *Cheilinus fasciatus* | 0.75 | 0.42 | 0.5 | 0.56 | Mollusk | Slow | Hard |
| *Cheilinus oxycephalus* | 1.17 | 0.5 | 0.47 | 0.47 | General Invertebrate | Intermediate | Soft |
| *Cheilinus trilobatus* | 0.79 | 0.39 | 0.48 | 0.51 | Mollusk, Echinoderm, Crab | Slow | Hard |
| *Cheilinus undulatus* | 0.75 | 0.37 | 0.61 | 0.5 | General Invertebrate | Slow | Hard |
| *Cheilio inermis* | 0.65 | 0.43 | 0.92 | 0.59 | Fish & Shrimp | Fast | Soft |
| *Choerodon anchorago* | 0.59 | 0.31 | 0.48 | 0.53 | Mollusks & Crustaceans | Slow | Hard |
| *Choerodon cephalotes* | 0.54 | 0.31 | 0.57 | 0.56 | Mollusk | Slow | Hard |
| *Choerodon cyanodus* | 0.64 | 0.35 | 0.48 | 0.54 | Mollusks & Crabs | Intermediate | Hard |
| *Choerodon fasciatus* | 0.8 | 0.37 | 0.43 | 0.49 | Mollusk | Slow | Hard |
| *Choerodon graphicus* | 0.6 | 0.35 | 0.44 | 0.57 | Mollusk & Crab | Intermediate | Hard |
| *Choerodon schoenleinii* | 0.66 | 0.34 | 0.52 | 0.51 | Mollusk | Slow | Hard |
| *Choerodon venustus* | 0.53 | 0.3 | 0.58 | 0.57 | Mollusk | Slow | Hard |
| *Clepticus parrae* | 1.95 | 0.68 | 0.61 | 0.36 | Zooplankton | Fast | Soft |
| *Coris aurilineata* | 0.69 | 0.38 | 0.52 | 0.55 | General Invertebrate | Intermediate | Soft |
| *Coris aygula* | 0.64 | 0.38 | 0.49 | 0.59 | Mollusk | Slow | Hard |
| *Coris batuensis* | 0.67 | 0.36 | 0.47 | 0.51 | Crab & Mollusk | Intermediate | Hard |
| *Coris dorsomacula* | 0.84 | 0.44 | 0.51 | 0.52 | General Invertebrate | Intermediate | Soft |
| *Coris gaimard* | 0.81 | 0.4 | 0.5 | 0.5 | Mollusk | Slow | Hard |
| *Coris pictoides* | 0.69 | 0.43 | 0.51 | 0.61 | General Invertebrate | Intermediate | Soft |
| *Cymolutes praetextatus* | 1.26 | 0.4 | 0.55 | 0.38 | General Invertebrate | Intermediate | Soft |
| *Cymolutes torquatus* | 1.03 | 0.41 | 0.52 | 0.4 | Zoobenthos | Slow | Soft |
| *Diproctacanthus xanthurus* | 0.76 | 0.4 | 0.59 | 0.53 | Coral Mucous | Slow | Soft |
| *Gomphosus varius* | 1.15 | 0.49 | 0.58 | 0.45 | Crab | Intermediate | Hard |
| *Halichoeres biocellatus* | 0.83 | 0.36 | 0.57 | 0.47 | General Invertebrate | Intermediate | Soft |
| *Halichoeres bivittatus* | 0.46 | 0.52 | 0.76 | 0.64 | Mollusk | Slow | Hard |
| *Halichoeres chloropterus* | 0.73 | 0.4 | 0.48 | 0.53 | General Invertebrate | Intermediate | Soft |
| *Halichoeres chrysus* | 0.73 | 0.41 | 0.58 | 0.54 | General Invertebrate | Intermediate | Soft |
| *Halichoeres garnoti* | 0.78 | 0.38 | 0.58 | 0.49 | Mollusk, Echinoderm, Crab, Echinoderm, Mollusk | Intermediate | Hard |
| *Halichoeres hortulanus* | 0.97 | 0.39 | 0.55 | 0.43 | Mollusk | Intermediate | Hard |
| *Halichoeres maculipinna* | 0.88 | 0.32 | 0.57 | 0.49 | Zoobenthos | Slow | Soft |
| *Halichoeres margaritaceus* | 0.71 | 0.36 | 0.5 | 0.51 | General Invertebrate | Intermediate | Soft |
| *Halichoeres marginatus* | 0.85 | 0.33 | 0.58 | 0.43 | General Invertebrate | Slow | Soft |
| *Halichoeres melanurus* | 0.8 | 0.38 | 0.53 | 0.48 | Zoobenthos | Slow | Soft |
| *Halichoeres melasmapomus* | 0.9 | 0.45 | 0.52 | 0.49 | Zoobenthos | Slow | Soft |
| *Halichoeres miniatus* | 0.79 | 0.42 | 0.48 | 0.53 | Zoobenthos | Slow | Soft |
| *Halichoeres nebulosus* | 0.63 | 0.38 | 0.44 | 0.6 | Mollusk | Slow | Hard |
| *Halichoeres nigrescens* | 0.9 | 0.42 | 0.51 | 0.47 | General Invertebrate | Intermediate | Soft |
| *Halichoeres ornatissimus* | 0.85 | 0.4 | 0.58 | 0.49 | General Invertebrate | Intermediate | Soft |
| *Halichoeres pictus* | 1.2 | 0.5 | 0.61 | 0.43 | Zoobenthos | Slow | Soft |
| *Halichoeres poeyi* | 0.84 | 0.39 | 0.58 | 0.46 | Mollusk, Echinoderm, Crab | Intermediate | Hard |
| *Halichoeres prosopeion* | 0.81 | 0.42 | 0.54 | 0.54 | General Invertebrate | Intermediate | Soft |
| *Halichoeres scapularis* | 0.9 | 0.42 | 0.53 | 0.48 | Zoobenthos | Slow | Soft |
| *Halichoeres trimaculatus* | 0.86 | 0.41 | 0.53 | 0.49 | Crabs & Mollusks | Intermediate | Hard |
| *Hemigymnus fasciatus* | 0.67 | 0.32 | 0.52 | 0.53 | Zoobenthos | Slow | Soft |
| *Hemigymnus melapterus* | 0.6 | 0.33 | 0.52 | 0.53 | Zoobenthos | Slow | Soft |
| *Hologymnosus annulatus* | 0.87 | 0.4 | 0.71 | 0.46 | Fish | Fast | Soft |
| *Hologymnosus doliatus* | 0.74 | 0.39 | 0.7 | 0.52 | Fish | Fast | Soft |
| *Labrichthys unilineatus* | 0.72 | 0.37 | 0.57 | 0.54 | Coral Mucous | Slow | Soft |
| *Labroides bicolor* | 0.55 | 0.4 | 0.74 | 0.61 | Cleaner | Slow | Soft |
| *Labroides dimidiatus* | 0.71 | 0.39 | 0.68 | 0.54 | Cleaner | Slow | Soft |
| *Labroides pectoralis* | 0.56 | 0.38 | 0.7 | 0.59 | Gnathiid | Slow | Soft |
| *Labropsis australis* | 0.6 | 0.35 | 0.59 | 0.56 | Coral Mucous | Slow | Soft |
| *Leptojulis cyanopleura* | 0.82 | 0.45 | 0.56 | 0.55 | Zooplankton | Fast | Soft |
| *Macropharyngodon choati* | 0.81 | 0.39 | 0.43 | 0.5 | Foraminifera | Slow | Hard |
| *Macropharyngodon kuiteri* | 0.9 | 0.41 | 0.48 | 0.47 | Foraminifera | Slow | Hard |
| *Macropharyngodon meleagris* | 0.67 | 0.32 | 0.44 | 0.48 | Foraminifera, Detritus, Mollusk | Slow | Hard |
| *Macropharyngodon negrosensis* | 0.84 | 0.39 | 0.45 | 0.49 | Foraminifera | Slow | Hard |
| *Novaculichthys taeniourus* | 0.87 | 0.39 | 0.46 | 0.49 | Brachyura Crabs | Intermediate | Hard |
| *Oxycheilinus bimaculatus* | 0.75 | 0.41 | 0.49 | 0.55 | General Invertebrate | Intermediate | Soft |
| *Oxycheilinus digramma* | 0.82 | 0.44 | 0.57 | 0.51 | Fish | Fast | Soft |
| *Oxycheilinus unifasciatus* | 0.73 | 0.4 | 0.55 | 0.53 | Fish | Fast | Soft |
| *Pseudocheilinus hexataenia* | 1.18 | 0.48 | 0.46 | 0.45 | Zoobenthos | Slow | Soft |
| *Pseudocheilinus octotaenia* | 0.83 | 0.49 | 0.49 | 0.55 | Brachyura Crabs | Intermediate | Hard |
| *Pseudocoris yamashiroi* | 0.92 | 0.53 | 0.54 | 0.57 | Zooplankton | Fast | Soft |
| *Pseudodax moluccanus* | 0.89 | 0.42 | 0.38 | 0.49 | Herbivore | Slow | Soft |
| *Pseudojuloides cerasinus* | 0.83 | 0.43 | 0.68 | 0.51 | Zoobenthos | Slow | Soft |
| *Pseudolabrus guentheri* | 0.76 | 0.39 | 0.47 | 0.54 | Zoobenthos | Slow | Soft |
| *Pteragogus cryptus* | 0.84 | 0.45 | 0.45 | 0.52 | Zoobenthos | Slow | Soft |
| *Stethojulis bandanensis* | 0.65 | 0.34 | 0.49 | 0.53 | Zoobenthos | Slow | Soft |
| *Stethojulis interrupta* | 0.7 | 0.39 | 0.55 | 0.54 | Zoobenthos | Slow | Soft |
| *Stethojulis strigiventer* | 0.55 | 0.44 | 0.6 | 0.64 | Zoobenthos | Slow | Soft |
| *Stethojulis trilineata* | 0.81 | 0.37 | 0.54 | 0.46 | Zooplankton | Fast | Soft |
| *Thalassoma amblycephalum* | 0.92 | 0.44 | 0.47 | 0.51 | Zooplankton | Fast | Soft |
| *Thalassoma bifasciatum* | 0.97 | 0.42 | 0.56 | 0.44 | Zooplankton | Fast | Soft |
| *Thalassoma hardwicke* | 0.89 | 0.41 | 0.52 | 0.46 | Crabs & Mollusk | Intermediate | Hard |
| *Thalassoma jansenii* | 0.86 | 0.38 | 0.51 | 0.45 | Crabs & Mollusks | Intermediate | Hard |
| *Thalassoma lunare* | 0.82 | 0.35 | 0.44 | 0.47 | Crabs & Mollusks | Intermediate | Hard |
| *Thalassoma lutescens* | 0.72 | 0.36 | 0.52 | 0.51 | Crabs & Mollusks | Intermediate | Hard |
| *Thalassoma quinquevittatum* | 0.87 | 0.39 | 0.53 | 0.47 | General Invertebrate | Intermediate | Soft |
| *Thalassoma trilobatum* | 0.64 | 0.37 | 0.47 | 0.56 | Crabs & Mollusks | Intermediate | Hard |
| *Wetmorella nigropinnata* | 1.11 | 0.57 | 0.57 | 0.48 | Zoobenthos | Slow | Soft |
| *Xyrichtys martinicensis* | 1.46 | 0.4 | 0.53 | 0.32 | Zoobenthos | Slow | Soft |
| *Xyrichtys novacula* | 1.18 | 0.34 | 0.57 | 0.33 | Mollusk, Echinoderm | Slow | Hard |
| *Xyrichtys splendens* | 1.25 | 0.41 | 0.61 | 0.36 | Zooplankton | Fast | Soft |

Supplementary Table 3: Summary of four-bar linkage information and dietary categorizations for cichlids. Four-bar data were adapted from Burress et al. (2020). Dietary data were collected from Burress (2016), Burress et al. (2019) and Burress et al. (2020). Details on how dietary categorizations were assigned can be found in the Methods.

| Species | KT | Input | Output | Coupler | Dietary Categorization | Velocity-Based Regime | Hard-Based Regime |
| --- | --- | --- | --- | --- | --- | --- | --- |
| *Acarichthys_heckelii* | 0.62 | 0.33 | 0.54 | 0.62 | Generalist | slow | soft |
| *Acaronia_nassa* | 0.43 | 0.32 | 0.72 | 0.39 | Predator | fast | soft |
| *Aequidens_diadema* | 0.60 | 0.40 | 0.54 | 0.54 | Generalist | slow | soft |
| *Aequidens_potaroensis* | 0.65 | 0.43 | 0.62 | 0.45 | Generalist | slow | soft |
| *Amatitlania_sajica* | 0.68 | 0.36 | 0.53 | 0.53 | Grazer | slow | hard |
| *Amatitlania_siquia* | 0.89 | 0.43 | 0.61 | 0.57 | Grazer | slow | hard |
| *Amphilophus_citrinellus* | 0.67 | 0.43 | 0.58 | 0.54 | Grazer | intermediate | hard |
| *Andinoacara_coeruleopunctatus* | 0.75 | 0.37 | 0.62 | 0.50 | Generalist | slow | soft |
| *Andinoacara_pulcher* | 0.73 | 0.40 | 0.48 | 0.72 | Generalist | slow | soft |
| *Apistogramma_agassizii* | 0.73 | 0.35 | 0.51 | 0.53 | Generalist | slow | soft |
| *Apistogramma_bitaeniata* | 0.58 | 0.36 | 0.62 | 0.45 | Generalist | slow | soft |
| *Apistogramma_cacatuoides* | 0.57 | 0.32 | 0.57 | 0.41 | Generalist | slow | soft |
| *Apistogramma_caetei* | 0.63 | 0.36 | 0.57 | 0.45 | Generalist | slow | soft |
| *Archocentrus_centrarchus* | 0.62 | 0.43 | 0.64 | 0.42 | Grazer | slow | soft |
| *Archocentrus_multispinosus* | 0.54 | 0.42 | 0.71 | 0.42 | Grazer | slow | hard |
| *Astronotus_ocellatus* | 0.67 | 0.36 | 0.54 | 0.51 | Generalist | slow | hard |
| *Biotodoma_cupido* | 0.52 | 0.35 | 0.65 | 0.34 | Generalist | slow | soft |
| *Caquetaia_kraussii* | 0.90 | 0.41 | 0.47 | 0.63 | Predator | fast | soft |
| *Caquetaia_myersi* | 0.56 | 0.43 | 0.68 | 0.36 | Predator | fast | soft |
| *Caquetaia_spectabilis* | 1.14 | 0.58 | 0.48 | 0.33 | Predator | fast | soft |
| *Caquetaia_umbrifera* | 0.66 | 0.49 | 0.64 | 0.36 | Predator | fast | soft |
| *Chaetobranchus_flavescens* | 0.59 | 0.46 | 0.69 | 0.42 | Generalist | slow | soft |
| *Cichla_kelberi* | 0.80 | 0.46 | 0.57 | 0.48 | Predator | fast | soft |
| *Cichla_monoculus* | 0.52 | 0.41 | 0.70 | 0.40 | Predator | fast | soft |
| *Cichla_ocellaris* | 0.49 | 0.38 | 0.71 | 0.41 | Predator | fast | soft |
| *Cichla_piquiti* | 0.71 | 0.44 | 0.59 | 0.35 | Predator | fast | soft |
| *Cichlasoma_bocourti* | 0.51 | 0.44 | 0.54 | 0.52 | Grazer | slow | hard |
| *Cichlasoma_grammodes* | 0.41 | 0.37 | 0.80 | 0.42 | Predator | intermediate | soft |
| *Cichlasoma_orientale* | 0.80 | 0.40 | 0.56 | 0.54 | Generalist | slow | soft |
| *Cichlasoma_urophthalmum* | 0.75 | 0.43 | 0.57 | 0.51 | Generalist | intermediate | soft |
| *Cincelichthys_pearsei* | 0.44 | 0.37 | 0.55 | 0.52 | Grazer | slow | soft |
| *Cleithracara_maronii* | 0.88 | 0.30 | 0.59 | 0.46 | Generalist | slow | soft |
| *Crenicara_punctulatum* | 0.70 | 0.49 | 0.55 | 0.49 | Generalist | slow | soft |
| *Crenicichla_alta* | 0.62 | 0.39 | 0.64 | 0.58 | Predator | fast | soft |
| *Crenicichla_lugubris* | 0.66 | 0.36 | 0.54 | 0.54 | Predator | fast | soft |
| *Crenicichla_reticulata* | 0.84 | 0.42 | 0.51 | 0.58 | Predator | fast | soft |
| *Cribroheros_robertsoni* | 0.55 | 0.48 | 0.79 | 0.54 | Sifter | slow | soft |
| *Cryptoheros_spilurus* | 0.92 | 0.57 | 0.64 | 0.62 | Grazer | slow | hard |
| *Geophagus_abalios* | 0.69 | 0.58 | 0.70 | 0.51 | Sifter | slow | soft |
| *Geophagus_brasiliensis* | 0.74 | 0.38 | 0.52 | 0.60 | Sifter | slow | soft |
| *Geophagus_steindachneri* | 0.70 | 0.35 | 0.50 | 0.51 | Sifter | slow | soft |
| *Geophagus_taeniopareius* | 0.73 | 0.39 | 0.57 | 0.70 | Sifter | slow | soft |
| *Guianacara_stergiosi* | 0.72 | 0.40 | 0.57 | 0.63 | Generalist | slow | soft |
| *Gymnogeophagus_balzanii* | 0.71 | 0.37 | 0.58 | 0.73 | Sifter | slow | soft |
| *Gymnogeophagus_labiatus* | 0.78 | 0.42 | 0.54 | 0.58 | Sifter | slow | soft |
| *Gymnogeophagus_meridionalis* | 0.64 | 0.33 | 0.53 | 0.64 | Sifter | slow | soft |
| *Herichthys_cyanoguttatus* | 0.71 | 0.37 | 0.54 | 0.63 | Grazer | intermediate | hard |
| *Herichthys_labridens* | 0.55 | 0.29 | 0.53 | 0.65 | Grazer | intermediate | hard |
| *Herichthys_steindachneri* | 0.80 | 0.38 | 0.51 | 0.65 | Grazer | intermediate | hard |
| *Heros_severus* | 0.61 | 0.33 | 0.54 | 0.53 | Grazer | slow | soft |
| *Hoplarchus_psittacus* | 0.71 | 0.40 | 0.57 | 0.56 | Generalist | slow | soft |
| *Hypselecara_temporalis* | 0.63 | 0.33 | 0.53 | 0.57 | Generalist | slow | soft |
| *Hypsophrys_nematopus* | 0.65 | 0.33 | 0.50 | 0.64 | Grazer | slow | hard |
| *Hypsophrys_nicaraguensis* | 0.70 | 0.37 | 0.52 | 0.55 | Grazer | slow | hard |
| *Laetacara_curviceps* | 0.66 | 0.43 | 0.64 | 0.50 | Generalist | slow | soft |
| *Laetacara_dorsigera* | 0.79 | 0.45 | 0.58 | 0.56 | Generalist | slow | soft |
| *Maskaheros_regani* | 0.73 | 0.44 | 0.60 | 0.53 | Grazer | slow | soft |
| *Mesonauta_egregius* | 0.89 | 0.48 | 0.54 | 0.52 | Grazer | slow | soft |
| *Mikrogeophagus_altispinosus* | 0.92 | 0.46 | 0.50 | 0.52 | Generalist | slow | soft |
| *Mikrogeophagus_ramirezi* | 0.77 | 0.45 | 0.58 | 0.53 | Generalist | slow | soft |
| *Nannacara_anomala* | 0.84 | 0.46 | 0.56 | 0.56 | Generalist | slow | soft |
| *Parachromis_dovii* | 0.72 | 0.40 | 0.55 | 0.46 | Predator | fast | soft |
| *Parachromis_friedrichsthalii* | 0.46 | 0.34 | 0.70 | 0.38 | Predator | fast | soft |
| *Parachromis_managuensis* | 0.67 | 0.39 | 0.57 | 0.38 | Predator | fast | soft |
| *Parachromis_motaguensis* | 0.49 | 0.37 | 0.69 | 0.34 | Predator | fast | soft |
| *Paraneetroplus_bifasciatus* | 0.82 | 0.45 | 0.56 | 0.59 | Grazer | slow | soft |
| *Paraneetroplus_fenestratus* | 0.58 | 0.31 | 0.53 | 0.58 | Grazer | slow | soft |
| *Paraneetroplus_hartwegi* | 0.65 | 0.36 | 0.55 | 0.55 | Grazer | slow | soft |
| *Paraneetroplus_maculicauda* | 0.66 | 0.39 | 0.58 | 0.46 | Grazer | slow | soft |
| *Petenia_splendida* | 0.63 | 0.42 | 0.63 | 0.38 | Predator | fast | soft |
| *Pterophyllum_scalare* | 0.76 | 0.41 | 0.54 | 0.51 | Grazer | intermediate | soft |
| *Retroculus_xinguensis* | 0.78 | 0.41 | 0.54 | 0.54 | Sifter | slow | soft |
| *Rheoheros_lentiginosus* | 0.82 | 0.45 | 0.56 | 0.54 | Grazer | slow | soft |
| *Rocio_octofasciata* | 0.67 | 0.37 | 0.55 | 0.52 | Generalist | intermediate | soft |
| *Satanoperca_daemon* | 0.55 | 0.52 | 0.71 | 0.39 | Sifter | slow | soft |
| *Satanoperca_jurupari* | 0.65 | 0.40 | 0.60 | 0.43 | Sifter | slow | soft |
| *Satanoperca_leucosticta* | 0.71 | 0.39 | 0.63 | 0.74 | Sifter | slow | soft |
| *Symphysodon_discus* | 0.86 | 0.49 | 0.58 | 0.56 | Grazer | slow | soft |
| *Theraps_irregularis* | 0.85 | 0.41 | 0.53 | 0.74 | Grazer | slow | soft |
| *Thorichthys_aureus* | 0.72 | 0.35 | 0.54 | 0.83 | Sifter | slow | soft |
| *Thorichthys_meeki* | 0.72 | 0.35 | 0.54 | 0.7 | Sifter | slow | soft |
| *Tomocichla_sieboldii* | 0.69 | 0.39 | 0.56 | 0.49758977 | Grazer | slow | soft |
| *Trichromis_salvini* | 0.68 | 0.26 | 0.45 | 0.42025012 | Generalist | intermediate | soft |
| *Uaru_amphiacanthoides* | 0.85 | 0.47 | 0.60 | 0.66029805 | Grazer | slow | soft |
